# Longitudinal analysis of SARS-CoV-2 infection and vaccination in the LA-SPARTA cohort reveals increased risk of infection in vaccinated Hispanic participants

**DOI:** 10.3389/fimmu.2023.1139915

**Published:** 2023-04-19

**Authors:** Meagan M. Jenkins, Donna Phan Tran, Evelyn A. Flores, Deborah Kupferwasser, Harry Pickering, Ying Zheng, David W. Gjertson, Ted M. Ross, Joanna M. Schaenman, Loren G. Miller, Michael R. Yeaman, Elaine F. Reed

**Affiliations:** ^1^ Department of Pathology and Laboratory Medicine, University of California Los Angeles, Los Angeles, CA, United States; ^2^ Lundquist Institute for Biomedical Innovation at Harbor–University of California, Los Angeles (UCLA) Medical Center, Torrance, CA, United States; ^3^ Department of Biostatistics, University of California Los Angeles, Los Angeles, CA, United States; ^4^ Center for Vaccines and Immunology, University of Georgia, Athens, GA, United States; ^5^ Department of Infectious Diseases, University of Georgia, Athens, GA, United States; ^6^ Department of Medicine, David Geffen School of Medicine at the University of California Los Angeles, Los Angeles, CA, United States; ^7^ Division of Infectious Diseases, Department of Medicine, Harbor-University of California, Los Angeles (UCLA) Medical Center, Torrance, CA, United States; ^8^ Division of Molecular Medicine, Harbor–University of California, Los Angeles (UCLA) Medical Center, Torrance, CA, United States; ^9^ Institute for Infection & Immunity, Lundquist Institute for Biomedical Innovation at Harbor–University of California, Los Angeles (UCLA) Medical Center, Torrance, CA, United States

**Keywords:** SARS-CoV-2, COVID-19, vaccination, infection, serological analysis, high-risk

## Abstract

**Introduction:**

SARS-CoV-2 is the etiologic agent of coronavirus disease 2019 (COVID-19). Questions remain regarding correlates of risk and immune protection against COVID-19.

**Methods:**

We prospectively enrolled 200 participants with a high risk of SARS-CoV-2 occupational exposure at a U.S. medical center between December 2020 and April 2022. Participant exposure risks, vaccination/infection status, and symptoms were followed longitudinally at 3, 6, and 12 months, with blood and saliva collection. Serological response to the SARS-CoV-2 spike holoprotein (S), receptor binding domain (RBD) and nucleocapsid proteins (NP) were quantified by ELISA assay.

**Results:**

Based on serology, 40 of 200 (20%) participants were infected. Healthcare and non-healthcare occupations had equivalent infection incidence. Only 79.5% of infected participants seroconverted for NP following infection, and 11.5% were unaware they had been infected. The antibody response to S was greater than to RBD. Hispanic ethnicity was associated with 2-fold greater incidence of infection despite vaccination in this cohort.

**Discussion:**

Overall, our findings demonstrate: 1) variability in the antibody response to SARS-CoV-2 infection despite similar exposure risk; 2) the concentration of binding antibody to the SARS-CoV-2 S or RBD proteins is not directly correlated with protection against infection in vaccinated individuals; and 3) determinants of infection risk include Hispanic ethnicity despite vaccination and similar occupational exposure.

## 1 Introduction

Severe Acute Respiratory Syndrome Coronavirus 2 (SARS-CoV-2) causes Coronavirus-Disease 2019 (COVID-19), a pandemic that emerged in December of 2019. Despite intensive worldwide investigations, specific determinants of risk or protection against SARS-CoV-2 infection and COVID-19 disease remain elusive. Furthermore, as viral variants continue to emerge, correlates of vulnerability and immunity have continued to evolve. Critical to gaining knowledge in this regard are key conceptual distinctions: 1) exposure *vs*. infection *vs*. disease; and 2) immune response *vs*. protective immune response *vs*. determinants of protective immunity.

Among diverse populations, SARS-CoV-2 exposure risks differ relative to several factors, including occupation, healthcare, lifestyle and household structure, among others. Limited information is available regarding SARS-CoV-2 infection, immune response and COVID-19 risk in frontline workers at urban medical centers who have among the greatest chance of exposure. Interestingly, Hancean et al. found that urban medical occupations do not significantly drive viral transmission as compared to non-medical professions (1). The tendency for individuals to associate with those who are similar to themselves (e.g. vaccinated individuals with other vaccinated individuals) has been hypothesized to explain such an observation. However, there is a paucity of information regarding occupation, vaccination or immune response relative to risks of SARS-CoV-2 exposure or infection in frontline healthcare workers.

Humoral immunity has long been correlated with vaccine efficacy and protection against infection, particularly where neutralizing antibody affords immunity against viral pathogens. The humoral response to SARS-CoV-2 vaccination has accordingly been linked to increased protection against infection (2, 3). However, the observed direct correlation between severity of COVID-19 disease and anti-RBD antibody titer (4–6), serious infection despite high vaccine induced anti-RBD antibody titers (7, 8), and broader immune protection arising from natural SARS-CoV-2 infection (9) suggests as yet unknown risks and antibody qualities contributing to outcomes.

During the study period, two SARS-CoV-2 vaccines were FDA-approved for use in the U.S. (Pfizer-BioNTech BNT162b2 or Comirnaty vaccine and Moderna mRNA-1273 or Spikevax vaccine), and one retained its Emergency Use Authorization (EUA) (Johnson & Johnson/Janssen JNJ-78436735 vaccine). However, SARS-CoV-2 continues to cause widespread global morbidity and mortality nearly two years after global vaccination efforts began. Asymptomatic viral carriers contribute to the challenge of infection control measures; it has been estimated that 40-45% of the globally infected individuals are asymptomatic (10, 11). Additionally, the emergence of viral variants has caused confusion to whether vaccine efficacy and knowledge gained based on those who have had natural infection with a previous variant will translate to subsequent variants.

Most serologic assays for SARS-CoV-2 assess either neutralizing or binding antibodies against the spike holoprotein (S), spike receptor binding domain (RBD), or nucleocapsid protein (NP). Serological response to the S protein can be detected in both vaccinated and naturally infected individuals. However, NP is not included in current vaccine formulations; therefore, only those individuals naturally infected by SARS-CoV-2 generate anti-NP responses. This fact allows for the detection of prior infection in individuals who may have been asymptomatic or otherwise unaware of infection.

From its onset, the COVID-19 pandemic has imposed a disproportionate impact on underserved populations in larger urbanized areas (12). Specifically, SARS-CoV-2 disproportionately affects racial and ethnic minority groups in the US (13–15). However, whether such disparities are due to socioeconomic, healthcare utilization, comorbidities, genetic or a combination of these and other factors is not yet clear. In Los Angeles, almost 50% of the infections occur in populations who identify as Hispanic (16). Early studies regarding the COVID-19 pandemic show that Hispanic participants in Los Angeles have a higher prevalence of infection with SARS-CoV-2, but as variants have emerged, there has been little follow up (17, 18). Thus, key questions remain regarding potential correlates of risk or immunity based on race or ethnicity.

To gain new insights and address potential correlates of COVID-19 risk and immunity in real-world context, here we serologically analyzed the Los Angeles cohort of the COVID-19 SeroPrevalence And Respiratory Tract Assessment (LA-SPARTA) study. The current study focused on high-risk individuals according to their occupation, antibody response to S, RBD, and NP proteins of SARS-CoV-2.

## 2 Materials and methods

### 2.1 SPARTA-LA study design

We prospectively enrolled 200 participants with a high risk of SARS-CoV-2 occupational exposure, irrespective of history of SARS-CoV-2 vaccination or natural infection. Participants were enrolled on the campuses of Harbor-UCLA Medical Center and The Lundquist Institute for Biomedical Innovation at Harbor-UCLA in Torrance, CA, between December 2021 and April 2021. Our cohort of participants in Los Angeles, CA is one of eight sites who enrolled participants starting in 2020 as part of PARIS (Protection Associated with Rapid Immunity to SARS-CoV-2)/SPARTA (SeroPrevalence and Respiratory Tract Assessment) for longitudinal analysis of SARS-COV-2 reinfection and correlates of protection. Previously, the cohorts of all PARIS and SPARTA cohorts, including a preliminary analysis of the LA-SPARTA cohort infections and collaborative efforts were reported (19).

### 2.2 Data collection/storage

Patients were recruited via flyers that were posted around the Harbor-UCLA campus (**Supplemental Figure 2**). Interested persons contacted a research coordinator and were screened for eligibility via interview. Study inclusion criteria were: age > 18 years of age, able to complete the informed consent process, willing/able to attend and complete scheduled study visits, and who fall within one of the following categories: full-time healthcare workers, work in the inpatient setting, and take care of patients with or at high risk of having COVID-19 infection (such as in the emergency department), or medical center full-time employees who do not have contact with persons with documented or suspected COVID-19 infection, or law enforcement who work full time while having contact with the general public, or paramedics or Emergency Medical Service (EMS) whose duties include full time interaction with patients, or other community members who are able to access the Harbor-UCLA campus. The exclusion criteria were: pregnancy, weight <110 lbs (50 kg), acute non COVID-19 infection, receipt of immunomodulatory or immune suppressive medication (e.g., chemotherapy, systemic steroids), in the prior 12 months, chronic infection (e.g. HIV, Hepatitis C), or conditions with immune dysregulation such as rheumatologic or autoimmune diseases.

Eligible participants gave informed consent and answered surveys to collect data on demographics, medical conditions, employment type, and symptoms of possible COVID infection. Participant height and weight were measured at enrollment and each follow up visit. At baseline, we performed saliva, nasal swab, and blood draws. Over the duration of the study period, participants were surveyed routinely to screen for additional vaccinations/boosters, infections, and health changes via weekly emails. If events were reported, such as vaccination or infection, participants would provide a blood and saliva sample in addition to samples that were collected at 3 months, 6 months, and 12 months after enrollment. Due to funding constraints, participants who had SARS-CoV-2 antibody responses at enrollment were prioritized to be sampled more often. Serological response against the SARS-CoV-2 Spike, RBD, and NP was detected via quantitative ELISA assay and analyzed accordingly. All survey and serological data were stored using the secure, REDCap database management software.

### 2.3 Quantitative ELISA assay

RBD and Spike Proteins were obtained from the central laboratory of the PARIS/SPARTA collaboration. 100 µL of proteins were coated onto a 96-well microplate at a concentration of 1µg/mL, 2µg/mL, or 0.5µg/mL of SARS-CoV-2 Receptor Binding Domain (RBD), Spike, or Nucleocapsid (NP) proteins, respectively and incubated overnight at 4°C. RBD and Spike proteins from the Wuhan-Hu-1 SARS-CoV-2 isolate were created by Florian Krammer’s lab at Mount Sinai (20) and produced and received from the Center for vaccines and immunology CORE Lab at UGA (accession# MT380724.1 and MT380725.1). The NP protein (Sino Biological cat# 40588-V08B) was constructed from the 2019-nCoV SARS-CoV-2 isolate (accession# YP_009724397.2). The concentration of IgG was determined using a previously described method (21, 22). Plates were rinsed three to five times with 0.1% tween 20 in PBS (0.1% PBST) when noted. Plates were blocked with 3% non-fat dry milk in 0.1% PBST for 1h at room temperature (RT). Patient plasma was heat inactivated for 45-60 minutes at 56°C and diluted to 1:120 in 1% non-fat dry milk in 0.1% PBST and added to the plate for 2h at RT or overnight at 4°C after rinsing five times with 0.1% PBST. Next, plates were similarly rinsed using 0.1% PBST and a 1:3000 dilution of goat anti-human IgG-HRP was added to the plates for 1h at RT. Plates were rinsed and developed with SigmaFast OPD tablets in PBS per the manufacturer’s instructions for 10 minutes. The reaction was stopped using 3M HCl and scanned at 492 and 700 nm using a BioTek Cytation 5 Microplate reader. The OD700 nm was subtracted from the OD492 nm and the averaged from triplicate runs for each patient sample.

The Standard Curve was run on each plate with patient samples using a concentration gradient of an S1 specific SARS-CoV-2 IgG1 Ab (AbCam cat# ab273073) for the RBD and Spike assays or NP IgG1 Ab (Invivogen cat# covn-mab1) for the NP assay. The log of each average OD492-700nm was logged and graphed and the linear equation was then calculated and applied to the samples to determine the concentration respective of the standard curve, corrected for the dilution factor, and converted from ng/mL to µg/mL.

The positive and negative values for the RBD, Spike, and NP ELISA assays were calculated using a mixtures model to define two distributions in the raw or Log2 transformed ELISA data (i.e., negative and positive) using package “mclust” in RStudio. The mean and standard deviation (SD) of the presumed-negative population was calculated and the seropositivity cutoff was set as 2 standard deviations above the mean of the presumed-negative population. The cutoff values for the RBD, Spike and NP ELISA assays were 0.383, 0.132, and 2.225 µg/mL, respectively.

### 2.4 Calculating the time since vaccination

Participants reported their SARS-CoV-2 vaccination dates via surveys at enrollment and were able to notify us through their surveys if they received a vaccination over the course of the study. If participants only reported the date of the first dose, a second dose date of 21 days after the first dose was recorded for Pfizer recipients and 28 days for Moderna recipients. When available, clinical coordinators were able to confirm vaccination dates with participants. In total, 7 participants were missing the date of their second vaccination dose, but had provided the first date. Time since vaccination and booster was calculated as the blood collection date minus the vaccination or booster date divided by 30.

### 2.5 Definitions

At enrollment, a participant with a positive detected NP or an RBD antibody response without receiving SARS-CoV-2 vaccination was classified at having a prior infection (**Supplemental Figure 1**). During the study, participants who reported a positive SARS-CoV-2 PCR or antigen test were also categorized as having a reported infection. For those who had a natural infection over the course of the study, we defined a “Breakthrough infection” as a serologically detected infection or reported infection detected by positive SARS-CoV-2 PCR or antigen test in a participant who has been vaccinated with two doses of an mRNA vaccine or single dose of the adenoviral vector-based vaccine. “Infection” was defined as a serologically detected infection or infection detected by positive SARS-CoV-2 PCR or antigen test in an unvaccinated participant. “Serologically detected infection” was defined as NP seroconversion (converting from a negative to positive concentration value) or a ≥4-fold increase in the serological NP concentration, when compared to the previous serological sample tested or RBD seroconversion (converting from a negative to positive concentration value) without a known vaccination event or a ≥4-fold increase in the serological RBD concentration compared to the previous serological sample tested. Re-infection was used to describe participants who had a serologically detected NP antibody response at enrollment (, suggesting a prior infection with SARS-CoV-2, and who were infected over the course of the study (including both reported and serologically detected infections). Serological response was defined as a 4-fold or higher increase in the concentration of antibody compared to the sample prior to when infection was detected or reported. No or minimal response was defined as no change in the antibody concentration or less than 4-fold increase in the antibody concentration in the infection sample compared to the sample prior to when infection was detected or reported. The 4-fold threshold was defined by the PARIS/SPARTA consortium to determine sero-positivity or possible infection (19).

### 2.6 Statistical analysis

For continuous measures, t-tests were used to compare variables between two groups or time frames. For categorical data, grouped analyses of data pre and post vaccination with prior infection were carried out using Fisher’s exact test or a T test was carried out comparing the delta change. Longitudinal comparisons of continuous and categorical variables between groups were carried out using a mixed model analysis. Comparisons of continuous and categorical variables (RBD after vaccination) in a single group were carried out using an ordinary one-way ANOVA with an appropriate multiple comparison’s test. For time-to-event data, an interval-censored Cox proportional hazards model was used to estimate relative risks of breakthrough since participants were surveyed periodically.

### 2.7 Analysis of decay rates

Visual inspection of plots of raw protein levels versus time since vaccination suggested that protein decay could be modeled exponentially. Thus, protein levels were log transformed to facilitate using a random-effects generalized least-squares (GLS) regression model for testing effects among protein type (RBD/Spike) and ethnicity (Hispanic/Non-Hispanic) in initial analyses of decay rates. Models included time-interaction terms and random-subject factors to account for repeated measures within the same individual. We used conventionally derived variance estimators for GLS regression and assumed asymptotic independence and normality of standard errors for statistical inferences.

Deeper inspection of the raw experimental data revealed that protein decay may behave according to a transition over two linear phases – an early and late phase post-vaccination. To estimate early (i.e., left slope)/late (i.e., right slope) decay rates and transition times (i.e., breakpoints), a second analysis was done using a piecewise linear regression model (23) fit to the raw protein levels stratified by ethnicity. Point estimates and their 95% confidence intervals were reported and used for statistical inferences assuming asymptotic approximations due to the lack of proper *a priori* hypotheses.

All statistical analyses were calculated via GraphPad Prism version 9.3.1 or Stata version 17, as needed. P-values were two-sided and judged statistically significant if less than a nominal type-1 error rate of 5%. Due to the study’s discovery nature, no additional multiplicity adjustments were made beyond the pairwise ANOVA comparisons listed above and no prediction validations were performed.

### 2.8 Study approval

The study was approved by Institutional Review Board of the University of California, Los Angeles (IRB#20-001649-AM-00009). Written informed consent was received by participants prior to study participation.

## 3 Results

### 3.1 Study population, rates of vaccination, and natural infection

The LA-SPARTA study population comprised 29.0% Asian, 57.5% White, 6.5% Black and 7.0% other races (**Table 1**). In the total participant population, 40.5% identified as Hispanic. The mean age was 40.2 years. The number of female participants was 139 (69.5%) and males were 61 (30.5%) (**Table 1**). At enrollment, 65.5% of the cohort was vaccinated with at least one dose of a SARS-CoV-2 mRNA or adenoviral vector vaccine. Pfizer-BioNTech (BNT162b2) vaccination predominated the vaccination type received by this cohort (**Table 2**). A history of prior natural infection in 26.5% of the study participants at enrollment was confirmed serologically via detectable NP or RBD antibody concentration (in participants who had not received a vaccination), (**Table 2**). The criteria for the initial serological grouping at enrollment are shown in **Supplemental Figure 1**. A total of 40 infections were detected over the course of the study period, which is described in detail in the next section. 50.7% of unvaccinated participants at enrollment underwent vaccination over the study period (**Table 2**). At the end of the study period, 83.0% of participants were vaccinated and 35.0% of participants had evidence of a prior infection (**Table 2**). Pfizer-BioNTech (BNT162b2) was the predominant vaccine received by LA-SPARTA participants, with 84.3% of participants receiving this vaccine by study completion.

**Table 1:** Participant demographic data.

All Participants (n=200)		
Age (Years)		
	Mean (SD)	40.24 ± 12.06
	Min, Max	19, 77
Gender, n (%)		
	Male	61 (30.5%)
	Female	139 (69.5%)
Race, n (%)		
	White	115 (57.5%)
	Asian	58 (29.0%)
	Black or African American	13 (6.5%)
	American Indian/Alaskan Native	1 (0.5%)
	Native Hawaiian or Pacific Islander	4 (2.0%)
	Multiple	8 (4.0%)
	Unknown	1 (0.5%)
Ethnicity, n (%)	Hispanic or Latino	81 (40.5%)
	Non-Hispanic or Latino	118 (59.0%)
	Unknown	1 (0.5%)
Exposure Category, n (%)		
	Medical Center/Healthcare Worker	101 (50.5%)
	Nurse	68 (67.3%)
	Physician	11 (10.9%)
	Respiratory Therapist	3 (3.0%)
	Radiology Technician	3 (3.0%)
	First Responder	2 (2.0%)
	Physical/Occupational Therapy	1 (1.0%)
	Other	13 (12.9%)
	Medical Center/Non-Healthcare Worker	44 (22.0%)
	Environmental Services	1 (2.3%)
	Facilities Management	5 (11.4%)
	Laboratory Staff	15 (34.1%)
	Social Worker/Case Manager	3 (6.8%)
	Other	20 (45.5%)
	Local Community Member/Research Staff	55 (54.5%)
	Lundquist Institute Employee	30 (54.5%)
	Person who lives nearby	25 (45.5%)

Data are represented as the number of participants (n) and the percentage of participants in the cohort or group (%), unless otherwise stated.

**Table 2:** Participant serological status at enrollment and over the study period.

All Participants (n=200)	
At Enrollment	
Vaccinated, n (%)	131 (65.5%)
Moderna (mRNA-1273), n (%)	20 (15.2%)
Pfizer (BNT162b2), n (%)	110 (84.0%)
J&J/Janssen (JNJ-78436735), n (%)	1 (0.8%)
Not Vaccinated, n (%)	69 (34.5%)
Prior Infection, n (%)	53 (26.5%)
No Prior Infection, n (%)	147 (73.5%)
Prior Vaccination and Infection, n (%)	25 (12.5%)
Prior Vaccination without Infection, n (%)	106 (53.0%)
No Vaccination with Infection, n (%)	28 (14.0%)
No Vaccination without Infection, n (%)	41 (20.5%)
Vaccination Over the Study Period	
Unvaccinated to Vaccinated, n (%)	35/69 (50.7%)
Moderna (mRNA-1273), n (%)	3 (8.6%)
Pfizer (BNT162b2), n (%)	30 (85.7%)
J&J/Janssen (JNJ-78436735), n (%)	2 (5.7%)
Received Booster Vaccination, n (%)	67/166 (40.4%)
At Study Completion	
Vaccinated, n (%)	166/200 (83.0%)
Moderna (mRNA-1273), n (%)	23 (13.9%)
Pfizer (BNT162b2), n (%)	140 (84.3%)
J&J/Janssen (JNJ-78436735), n (%)	3 (1.8%)
Not Vaccinated, n (%)	29/200 (14.5%)
Unknown, n (%)	5/200 (2.5%)
Prior Infection, n (%)	70/200 (35.0%)
No Prior Infection, n (%)	120/200 (60.00%)
Unknown, n (%)	10/200 (5.0%)

Data are represented as the number of participants (n) and the percentage of participants in the cohort or group (%), unless otherwise stated. The number of participants who were vaccinated and infected at study completion is representative of each participant while they were in the study.

### 3.2 Serological assessment of SARS-CoV-2 infection in participants

Plasma was assessed at enrollment for antibody against S, RBD and NP proteins of the SARS-CoV-2 coronavirus. Participants were further stratified based on antibody reactivity to RBD and NP proteins (**Supplemental Figure 1**). We observed 6 unvaccinated participants with RBD seroconversion but without NP seroconversion at enrollment (**Table 3**). This pattern of results suggested these participants had been naturally infected due to evidence of seroconversion to RBD but not NP (**Supplemental Figure 1**). Accordingly, the criteria for assessing infection throughout the study period included NP seroconversion (defined as converting from a negative to positive concentration value if the value was not already within the positive range) or a ≥4-fold increase in NP concentration compared to the previous serological sample tested in previously positive individuals, or RBD seroconversion without a known vaccination event (converting from a negative to positive concentration value if the value was not already within the positive range) or a ≥4-fold increase in RBD concentration compared to the previous serological sample tested in previously positive individuals.

**Table 3:** Serological response to infection based on RBD and NP antibody response.

LA-SPARTA Infections			
Previous Infections at Enrollment, n (%)	53 (26.5%)		
NP Seroconversion, n (%)	47 (88.7%)		
RBD Seroconversion, n (%)	49 (92.5%)		
NP & RBD Seroconversion, n (%)	43 (81.1%)		
Infections During the Study Period			
	**Reported**	**Unreported**	**Total**
Infection in un-vaccinated person, n (%)	2 (1.0%)	8 (4.0%)	10 (5.0%)
Re-Infection, n	1	4	5
NP response, no/minimal RBD response, n	1	1	2
RBD response, no/minimal NP response, n	0	4	4
Minimal Serological Response, n	1	0	1
RBD & NP response, n	0	3	3
Breakthrough or Infection in Vaccinated Person, n (%)	15 (7.5%)	15 (7.5%)	30 (15.0%)
With serological Data, n	14	15	29
Re-Infection, n	4	6	10
NP response, no/minimal RBD response, n	5	8	13
RBD response, no/minimal NP response, n	0	2	2
Minimal Serological Response, n	2	0	2
RBD & NP response, n	7	5	12

Data are represented as the number of participants (n) and the percentage of participants in the cohort or group (%), unless otherwise stated.

**Figure 1:**
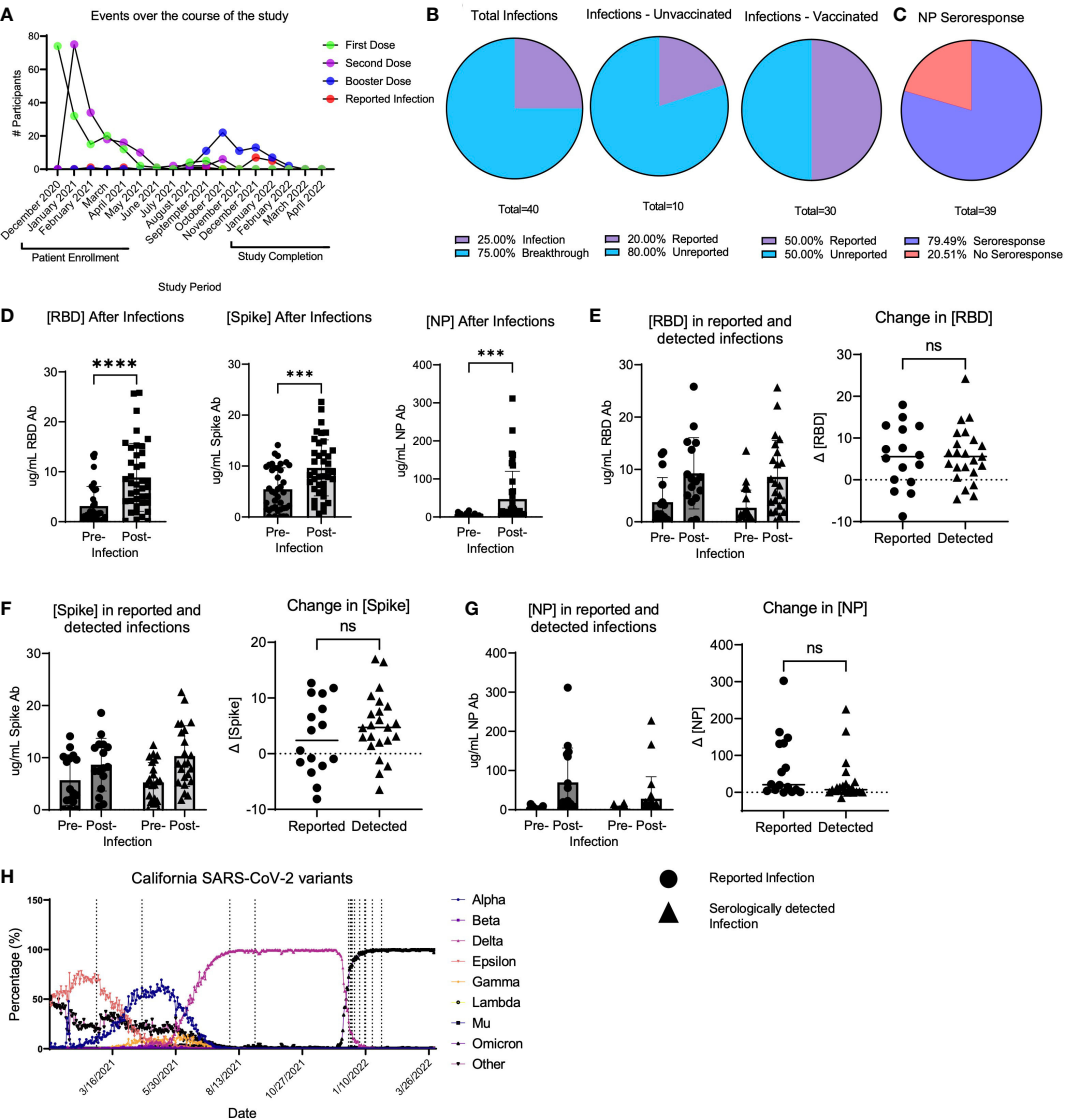
LA-SPARTA infections during the study period. **(A)** shows the events that occurred for all LA-SPARTA participants over the course of the study period. **(B)** describes both the reported and serologically detected infections in unvaccinated participants and breakthrough infections in vaccinated participants. **(C)** shows the percentage of infections and breakthrough infections in which the NP concentration increases by ≥4-fold compared to the previous blood sample collected. **(D)** shows the concentration of RBD, Spike, and NP in all serologically detected and reported infections and breakthrough infections. **(E)** shows the concentration of RBD in infections and breakthrough infections, separated by serologically detected versus reported infections. The delta change between the pre-infection sample collected and post-infection samples is also shown. **(F)** shows the concentration of Spike in infections and breakthrough infections, separated by serologically detected versus reported infections. The delta change between the pre-infection sample collected and post-infection samples is also shown. **(G)** shows the concentration of NP in infections and breakthrough infections, separated by serologically detected versus reported infections. The delta change between the pre-infection sample collected and post-infection samples is also shown. **(H)** uses SARS-CoV-2 variant data available from the California department of Health and Human Services from Los Angeles County to illustrate the percentage of each variant circulating at the time of reported positive tests (vertical dashed lines) in the LA-SPARTA cohort. *** designates P >0.001 and **** designates P >0.0001. ns, not significant.

Over the observation period, 40 total participants either reported a SARS-CoV-2 infection or had a natural infection detected serologically. Out of all infections, 10/40 (25.0%) occurred in unvaccinated participants, while 30/40 (75.0%) infections occurred using the same criteria in vaccinated individuals (termed “breakthrough infection”) (**Figures 1A, B**). It is important to note that the increased number of infections in vaccinated participants is likely because over the course of the study, the study population increased from 65.5% to 83.0% of participants vaccinated. Thus, 20.0% of the study cohort was infected with SARS-CoV-2 during the study period. Among these infections, 8 were unreported in unvaccinated participants, and 15 infections were unreported in vaccinated participants (**Figure 1B**). Of all participants who either reported a positive polymerase chain reaction (PCR) or antigen test over the course of the study (termed “reported infections”) and those who had serological evidence of infection (termed “unreported” or “serologically detected” infection), 20.5% of participants had no evidence of NP seroconversion (**Figure 1C**). In all infections, including those reported and serologically detected, there were post-infection increases in the concentrations of RBD, S, or NP antibodies in the plasma (**Figure 1D**). However, not all infections yielded an RBD response of ≥ 4-fold increase in concentration (**Table 3**). The RBD-, S-, or NP-specific serological responses did not significantly differ between reported infections and those unreported but detected serologically (**Figures 1E–G**). Many of the reported infections during the study period took place as the Omicron SARS-CoV-2 variant began to circulate in California, according to data from the California department for Health and Human Services (24) (**Figure 1H**). However, it is unclear if this is also true for the serologically detected infections, as we were unable to estimate the timing of the serologically detected infections due to a lack of positive test date.

### 3.3 SARS-CoV-2 vaccination response differs between RBD and non-RBD antibodies

**Figure 2:**
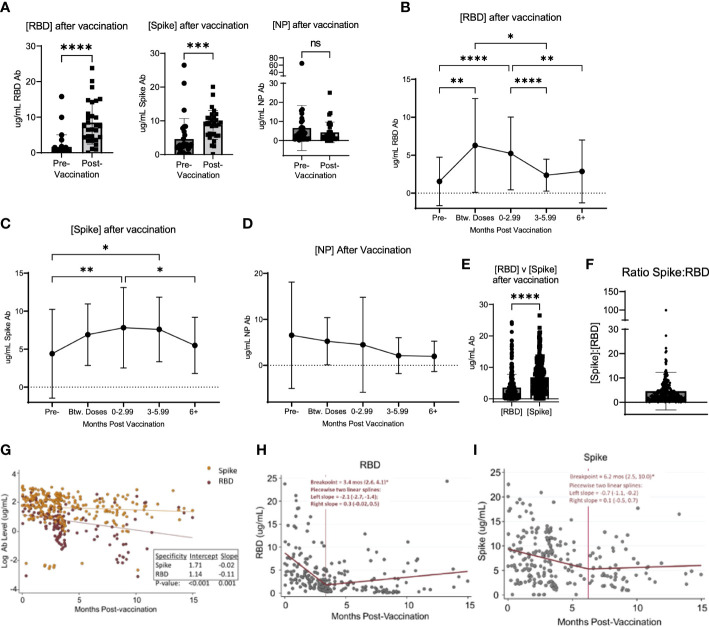
Vaccination induced changed in serological response to SARS-CoV-2. **(A)** shows the change in the concentration of RBD, Spike, and NP in participants who were vaccinated over the course of the study. **(B)** shows the change in RBD, Spike **(C)**, and NP **(D)** concentration after vaccination in all participants, regardless of when participants were vaccinated and when their blood was collected after vaccination. **(E)** shows the value of [RBD] and [Spike] in all participants after vaccination. **(F)** shows the ratio of the concentration of RBD to Spike for all participants after vaccination. **(G)** shows the log-transformed RBD (red data points) and Spike (orange data points) concentrations (i.e., to be consistent with exponential decay) over time using a GLS regression model **(H)** The lines represent the decay lines. Panel **(H)** shows the plot generated from piecewise linear regression models for the RBD protein or Spike protein **(I)**. The vertical line represents the “breakpoint”, which is the estimate of when the two linear phases of raw decay change. The two decay phases are shown before and after each breakpoint. The breakpoint and decay slopes for the left and right portion are noted in the graphs. * designates P >0.05, ** designates P >0.01, *** designates P >0.001, and **** designates P >0.0001. ns, not significant.

Of the unvaccinated participants at enrollment, 50.7% received a SARS-CoV-2 vaccination over the course of the study period (**Table 2**). At study completion, 83.0% of the study participants had received at least 2 doses of an mRNA-based or a single dose of an adenoviral vector-based SARS-CoV-2 vaccine (**Table 2**). Among participants who had samples collected both before and after vaccination, the concentration of RBD and S antibodies significantly increased after vaccination, while the concentration of NP antibodies did not significantly change (**Figure 2A**). To determine the kinetics of the binding antibody response to SARS-CoV-2 vaccination, confounding samples were censored (e.g. those collected after a booster vaccination or infection) and the remaining longitudinal samples were analyzed post-vaccination. This analysis revealed a response to vaccination that differed between RBD- and S-specific responses (**Figures 2B, C**). RBD-specific antibodies appeared to wane more rapidly than S-specific responses after vaccination, with RBD antibodies significantly decreasing by 3 months (**Figure 2B**) post-vaccination as compared to S responses which did not significantly decrease until ≥ 6 months (**Figure 2C**). There was no significant change in the NP antibody concentration after vaccination (**Figure 2D**). Accordingly, S antibody responses were higher than RBD antibody responses after vaccination (**Figure 2E**). The average ratio of RBD to S antibodies was 4.56 ± 7.7 (SD) µg/mL following vaccination (**Figure 2F**).

To compare the kinetics of RBD and S antibody decay, we first analyzed log-transformed RBD and S antibody levels (i.e., consistent with exponential decay) over time using a Generalized Least Squares regression model (**Figure 2G**). The constant decay rates (expressed as log ug/mL values) were -0.11 units/month and -0.02 units/month for anti-RBD and anti-S antibody, respectively. These two rates were significantly different (p=0.001). Also, average initial antibody levels (i.e., the estimated intercepts) were higher versus S than RBD, with initial values of 5.5 ug/mL (e^1.71) and 3.1 ug/mL (e^1.14), respectively (p<0.001). Next, we applied piecewise linear regression models separately for each antibody target to compare time estimates of two linear phases of raw decay change, or the “breakpoint”. Accordingly, the concentration of RBD antibodies decayed at a rate (left slope) of -2.1 (95% CI: -2.7, -1.4) ug/mL/month for 3.4 months (95% CI: 2.6, 4.1) and then decreased to 0.3 (95% CI: -0.02, 0.5) ug/mL/month as indicated by the slope thereafter (**Figure 2H**). Notably, after the breakpoint at 3.4 months, an *ad hoc* hypothesis test that decay ceases (i.e., the right slope equals zero) was not rejected at a 5% error rate since the 95% CI lower bound is negative and the upper bound is positive. For S protein, the concentration of antibodies decreased at a rate of -0.7 (95% CI: -1.1, -0.2) ug/mL/month for 6.2 months (95% CI: 2.5, 10.0) and then 0.1 (05% CI: -0.5, 0.7) ug/mL/month thereafter (**Figure 2I**). Similarly, after the breakpoint at 6.2 months, the *ad hoc* hypothesis that the antibody level was constant was not rejected.

### 3.4 Vaccination response differs in participants with prior infection

**Figure 3:**
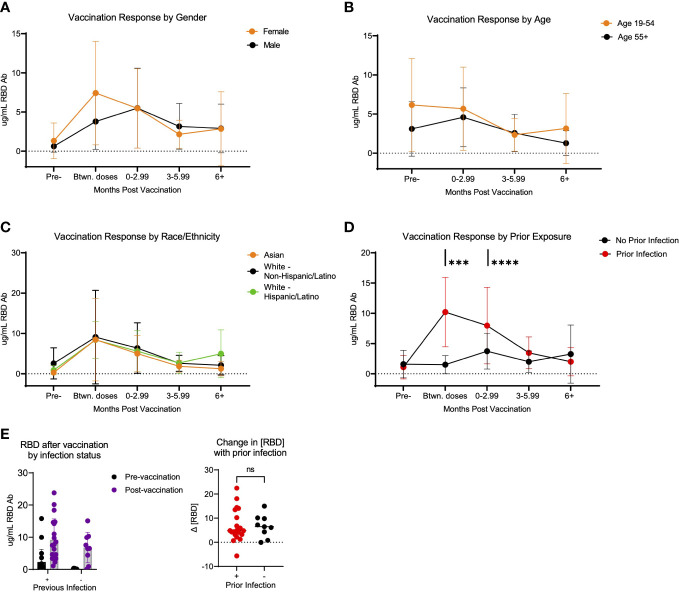
Response to SARS-CoV-2 vaccination in LA-SPARTA participants differs with prior infection. **(A)** shows the concentration of RBD over time according to age. Participants over 55 years of age were compared to all participants below 55 years of age. **(B)** shows the concentration of RBD over time according to gender. **(C)** shows the concentration of RBD over time according to race/ethnicity. **(D)** shows the concentration of RBD over time according to prior infection status before vaccination. **(E)** shows only the difference in RBD concentration in participants who were vaccinated over the course of the study, using the first available sample after vaccination (regardless of timing after vaccination) according to whether they had a prior infection at enrollment and prior to vaccination. The change in RBD concentration is also shown between the pre-infection and post-infection blood samples. *** designates P >0.001 and **** designates P >0.0001. ns, not significant.

To analyze potential differences in the response to vaccination based on the concentration of RBD specific antibodies, we compared antibody concentrations in various groups of participants following vaccination. Using the same method of eliminating confounding samples described above, we analyzed whether differences were detectable in the response to vaccination based on the concentration of RBD specific antibodies. Antibodies specific to the SARS-CoV-2 RBD protein have been shown to be specific to this virus, as the RBD domain is poorly conserved among other members of the SARS-coronavirus clade and other common coronaviruses (25–27). Thus, we focused on RBD-specific responses, as it is the key target of SARS-CoV-2 vaccines and binding RBD antibodies are associated with strong neutralizing SARS-CoV-2 responses (28, 29). To assess potential demographic correlates (age, ethnicity, and biological sex) that may influence the SARS-CoV-2 vaccination-induced antibody response, we compared the kinetic responses stratified to these variables. No significant differences in the binding antibody response to SARS-CoV-2 vaccination occurred according to age, ethnicity, or sex in this cohort (**Figures 3A–C**). There was however a significant difference based on whether the participants had a prior infection detected serologically. This difference occurred in the early vaccination response, noted at the time in between the 1st and 2nd vaccination dose (p=0.0003) and after 0-2.99 months post-vaccination (p<0.0001) (**Figure 3D**). Importantly, this difference in response was short lived, as no significant difference was detected in the response at ≥3 months post-vaccination, based on prior infection status (p=0.441 at 3-5.99 months post-vaccination and p=0.7532 at >6 months post-vaccination) (**Figure 3D**). Using this same dataset, we also analyzed the pre-and post- vaccination response in all participants who were vaccinated over the course of the study, using the first sample post-vaccination, without separating the data by time post vaccination. There was no significant difference in the change in antibody response when comparing participants with *vs*. without prior infection (p=0.9234) (**Figure 3E**).

### 3.5 Factors associated with breakthrough SARS-CoV-2 infection

**Figure 4:**
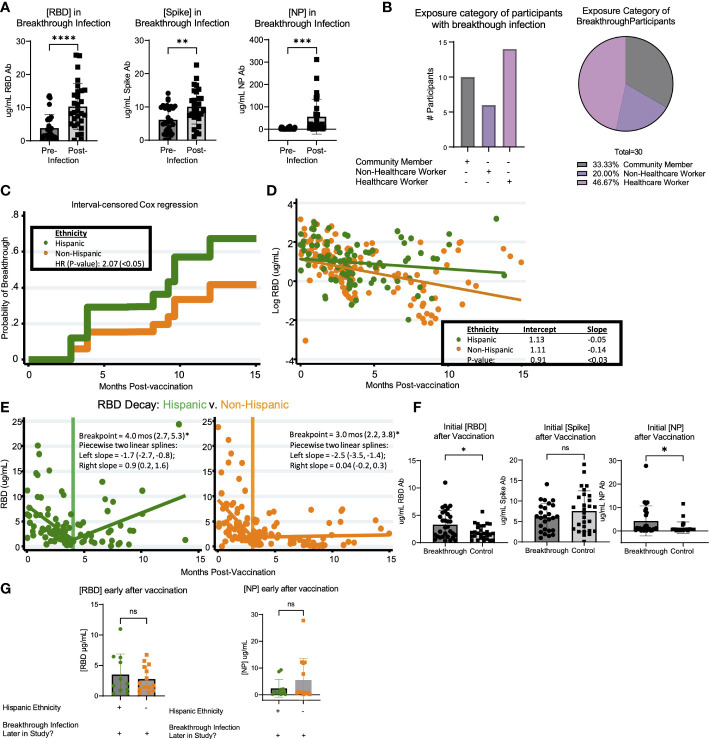
Breakthrough infection in vaccinated LA-SPARTA participants is more prevalent in Hispanic participants. **(A)** shows the change in RBD, Spike, and NP concentration between the reported and serologically detected breakthrough infections at the prior blood sample before the breakthrough infection was either reported or detected and after. **(B)** shows the distribution of risk category in the participants with breakthrough infections by number (left) and percentage (right). **(C)** An interval-censored regression model was used to compare the hazard ratios for breakthrough infection between Hispanic and Non-Hispanic participants. The Kaplan Meier curve is shown to represent this analysis. **(D)** shows the decay rate for antibodies against both RBD and Spike proteins on the log scale between Hispanic and non-Hispanic vaccinated participants. Panel **(E)** shows the piecewise linear regression model to find times at which the slopes of raw values change among Hispanic (left plot) and non-Hispanic participants (right plot). This analysis included 65 Hispanic participants, who contributed 95 samples and 93 non-Hispanic participants, who contributed 148 samples to the analysis. **(F)** shows the concentration of RBD, Spike, and NP after roughly 1-4 months after vaccination, in participants who had breakthrough infection later in the study and control participants who did not have breakthrough infection. **(G)** shows the concentration of RBD and NP after roughly 1-4 months after vaccination, in participants who had breakthrough infection later in the study according to ethnicity.* designates P >0.05, ** designates P >0.01, *** designates P >0.001, and **** designates P >0.0001. ns, not significant.

Breakthrough infection occurred in 30 of 166 (18.1%) of the vaccinated participants over the course of the study. Thus, we sought to determine if there were correlates associated with greater prevalence of breakthrough infection. Based on cumulative breakthrough infections in the study cohort, there was a significant increase in the concentration of RBD-, S- and NP- specific antibodies after infection as compared to infection naive (**Figure 4A**). Interestingly, there was an equivalent incidence of community members (10 participants) and healthcare workers (14 participants) who had breakthrough infection in this cohort (**Figure 4B**). To compare the variables that may be associated with a higher risk of breakthrough infection, we compared breakthrough participants with control participants who had been vaccinated against SARS-CoV-2 but did not have breakthrough infection over the course of this study. The demographic characteristics of these populations are shown in **Table 4**. Analysis of these data revealed a trend of increased incidences of infection among participants self-identifying as having Hispanic ethnicity in the breakthrough participant group compared to the control group (p=0.19; **Table 4**). Therefore, we next examined the possibility that Hispanic participants have a disproportionate risk of breakthrough infection with SARS-CoV-2 relative to time since vaccination. As shown in **Figure 4C**, we estimated probabilities of breakthrough infection as the time post-vaccination increases using an interval-censored Cox Regression model. This approach revealed that Hispanic participants exhibited twice the relative risk of breakthrough infection compared with non-Hispanic participants (hazard ratio=2.07; p< 0.05). We attempted to compare breakthrough risk among non-White participants but we only had sufficient study subject numbers to analyze Asian versus non-Asian participants. Comparison of the incidence of breakthrough infection in Asian versus non-Asian participants there was no significant difference in the breakthrough risk (data not shown).

**Table 4:** Breakthrough and vaccinated control participant demographic comparison.

	Total (n=60)	Breakthrough (n=30)	Control (n=30)	p-value
**Age (years), mean ± SD**	41 ± 12	41 ± 12	41 ± 12	0.96
**Gender, n (%)**				1.00
Female	44 (73%)	22 (73%)	22 (73%)	
Male	16 (27%)	8 (28%)	8 (27%)	
**Race, n (%)**				0.28
Asian	18 (30%)	7 (23%)	11 (37%)	
Black/African American	3 (5%)	2 (7%)	1 (3%)	
White/Caucasian	35 (58%)	19 (63%)	16 (53%)	
Other	4 (7%)	2 (7%)	2 (7%)	
**Ethnicity, n (%)**				0.19
Hispanic/Latino	24 (40%)	15 (50%)	9 (30%)	
Non-Hispanic/Latino	36 (60%)	15 (50%)	21 (70%)	
**BMI, mean ± SD**	29 ± 7	30 ± 7	28 ± 8	0.33
**Exposure, n (%)**				0.65
Medical Center/Healthcare Worker	25 (42%)	14 (47%)	11 (37%)	
Medical Center/Non-Healthcare Worker	15 (25%)	6 (20%)	9 (30%)	
Community Member/Research Staff	20 (33%)	10 (33%)	10 (33%)	

T-test was used to compare continuous variables such as age and BMI. Fisher’s Exact test was used for comparing categorical variables such as gender, race, and ethnicity. Data are represented as the number of participants (n) and the percentage of participants in the cohort or group (%), unless otherwise stated.

Additionally, we compared the logarithmic-scale decay rate for antibodies against RBD and S proteins between vaccinated Hispanics and non-Hispanics. Although initial RBD antibody levels were statistically equivalent, the RBD decay rates (expressed as log ug/mL values) were -0.05 units/month and -0.14 units/month for Hispanic and Non-Hispanic subjects, respectively (**Figure 4D**). These two rates were significantly different (p<0.03). By comparison, while initial S antibody levels were higher among Hispanic participants (7.3 ug/mL *vs*. 4.6 ug/mL, p<0.03), their logarithmic decay rates were indistinguishable from one another (data not shown). Given the overall difference in log-RBD antibody decay slopes, we used piecewise linear regression to find times at which the slopes of raw values changed. Here, we found similar temporal patterns and overlapping confidence intervals among Hispanics and non-Hispanics (**Figure 4E**). Finally, we compared the initial response against SARS-CoV-2 vaccination relative to concentration of anti-RBD, S, and NP antibodies within the first 4 months after vaccination. Results suggested a statistically significant increase in the early concentration of RBD antibodies after vaccination in the breakthrough infection group (p=0.0493); however, these individuals also had higher concentrations of anti-NP antibodies (p=0.0261) (**Figure 4F**). These findings suggested that the higher antibody response to vaccination was associated with having SARS-CoV-2 infection prior to vaccination. We analyzed the incidence of previous infection at enrollment when comparing Hispanic (28/53) versus non-Hispanic (25/93) participants and found that there was a relationship between ethnicity and prior infection (p=0.0496). When comparing the population of participants who had breakthrough infection, the early concentration of RBD and NP antibodies after vaccination were not significantly different between Hispanic and non-Hispanic participants (**Figure 4G**). Taken together, these results suggest that there may not be a direct quantitative link between early antibody response and breakthrough infection, or longer-term antibody kinetics (persistence or decay) and breakthrough infection.

## 4 Discussion

The results of this study illustrate differential exposure and immune response patterns to SARS-CoV-2 among a diverse population of high-risk individuals in the urban LA-SPARTA cohort from December 2020 – April 2022. This time period encompasses a major shift in SARS-CoV-2 variant emergence from alpha and beta, to delta to omicron lineages. Using a strategic serological approach, studies were designed to detect response to vaccination as well as previous natural SARS-CoV-2 infections. We identified a significant proportion of participants who experienced one or more SARS-CoV-2 infections over the course of the study. Of note, the emergence of the omicron variant was temporally associated with a significant increase in the number of reported SARS-CoV-2 infections. Many of these infections were detected serologically, without symptoms or illness reported by the participant.

Importantly, our results suggest that confirmatory detection of SARS-CoV-2 infection may be best based on both seroresponse against RBD and NP proteins. Others have noted that anti-NP seroconversion does not occur in all naturally-infected individuals (30). Our observation regarding the decreased NP seroresponse compared to RBD agrees with such studies, in which SARS-CoV-2 PCR positive participants may be non-responders to NP protein. To our knowledge, the present study is the first to directly report humoral responsiveness to RBD, but not NP after natural infection (31, 32). Other studies in which all participants achieved seroconversion have hypothesized that a decrease in viral RNA copy number or magnitude of symptoms are associated with a decreased anti-NP seroconversion (33). Parallel studies have also noted that lower Ct values via SARS-CoV-2 PCR testing are associated with lower seroconversion for S or NP proteins (34).

Collectively, the present results suggest that participants with prior infection had a stronger and more rapid response to the SARS-CoV-2 vaccination as compared to infection-naïve vaccinees. However, this effect did not impact the longer-term RBD-specific antibody response and was not associated with relative risk of exposure based on occupation. Notably, when determining whether there was a significant change in the pre- to post- vaccination antibody concentration respective of infection status, we found no significant correlates over the course of the study. This result may be due to the smaller sample size in those who were vaccinated.

We found that the RBD-specific antibody response to SARS-CoV-2 vaccination was significantly decreased by 3 months post-vaccination regardless of occupation or exposure risk. The antibody-based threshold of protection is unknown and antibody alone is unlikely to afford full protection against SARS-CoV-2 infection or COVID-19 disease. It is reasonable to hypothesize that even though decreased, antibody response can still contribute to protection in vaccinated individuals. Conceivably, quality of antibody in neutralizing virus to prevent or mitigate pathogenesis may be as or perhaps more important than quantity. Supporting this concept are several lines of evidence. In a large cohort study (SIREN) in the United Kingdom, while protection against infection was 72-92% efficacy after ~2 months post-vaccination, protection remained at 22–69% efficacy at 6 months post-vaccination despite a significant drop in antibody titer (35). Additionally, a recent meta-analysis found that protection against infection decreased by 20-30% after 6 months, however, protection against severe COVID-19 disease decreased by only 10% at 6 months post-vaccination (36). However, neither reports showed any corresponding antibody concentrations that correlate with protection from infection. Lastly, patients suffering the greatest frequency of infection or most severe COVID-19 disease can have among the highest titers (4, 5). Thus, qualitative protection may supersede absolute quantity of antibody response.

Of the total population in the current study, 20.0% were infected over the course of follow up, according to both reported infections and serological detection of infected individuals. Of the infections that occurred during the study period, 75% were in vaccinated participants and 25% in unvaccinated participants. However, it is important to note that 65.5% of participants were vaccinated at enrollment and the vaccination frequency increased over the course of the study, such that 83.0% of participants were vaccinated by study completion. Taking this into account, 30 out of 166 (18.0%) of vaccinated participants had breakthrough infection, whereas 10 of 29 (34.5%) unvaccinated participants were infected. Additionally, it is important to note that all infections that occurred during this study were mild and many may have even been asymptomatic based on the absence of reported participant symptom data on their surveys. Additionally, interpretation should be tempered by the fact that efficacy in terms of protection against infection and severe disease differs across studies.

Analysis of the cohort who had breakthrough infection during the study period despite vaccination revealed several interesting observations. First, healthcare workers who are at high risk for occupational exposure to SARS-CoV-2 did not appear to be at a higher risk for breakthrough infection in our cohort. Second, Hispanic participants exhibited twice the relative risk of breakthrough infection compared with non-Hispanic participants. Third, the antibody concentration early after vaccination did not appear to be predictive of breakthrough infection, nor did the concentration of antibodies prior to breakthrough infection. This may suggest other determinates, such as molecular (e.g. interferons) and cellular (e.g. CD8+ T cells) effectors or mucosal immunity may be integral to protective immunity. There may also be other factors such as how different aspects of the immune system interact to mediate protection against different SARS-CoV-2 variants. For example, mucosal IgA generated by WT or earlier SARS-CoV-2 variant infections may be more protective against later variants such as Omicron (37). Thus, additional correlates of protection against breakthrough infection should be studied. Additionally, it is possible that participants were infected with different SARS-CoV-2 variants, against which the serological response to the RBD and S of the Wuhan-Hu-1 SARS-CoV-2 isolate may not be as protective.

A recent study showed that in a population of adults in Chicago, Hispanic participants were at higher risk for infection with SARS-CoV-2 than non-Hispanic participants (14). Comparatively, our results show that Hispanic participants may be at higher risk of SARS-CoV-2 breakthrough infection. Interestingly, 28 (52.8%) Hispanic and 25 (47.2%) Non-Hispanic participants were infected at enrollment. Recent reports show that COVID-19 has disproportionately affected racial and ethnic minorities in terms of infection, hospitalizations and deaths with Hispanics having worse outcomes than non-Hispanics (16, 38). While the causes of racial and ethnic COVID-19 disparities remain unclear, there may be many reasons for this observed effect including socioeconomic factors, multigenerational households, or immunogenetic differences. We did not collect data on these factors and our cohort size deters us from inferring any possible differences based on these factors. Thus, additional studies with a larger population size are warranted to investigate these differences in SARS-CoV-2 infection and breakthrough infection in this population.

The limitations of our study include the relatively small number of participants, the absence of a positive SARS-CoV-2 PCR test for all infected participants, and the range in time for sample collection after vaccination for vaccinated participants. Thus, our data regarding the difference in SARS-CoV-2 infection in Hispanic participants should be validated in a larger cohort of participants. In addition, we did not require that our participants provide proof of a positive SARS-CoV-2 PCR or antigen test when testing for SARS-CoV-2 infection. We did, however, screen the participants at enrollment for serological evidence of prior infection. Using the criteria in this study it is possible to have missed participants with a prior infection if they had been vaccinated, as the NP response has been shown to decrease faster than that of S (39). Thus, if a participant was vaccinated, we would only be able to base our determination of prior infection on the NP antibody concentration which may be below the positivity threshold, depending on when the infection took place. We were also unable to determine whether there were differences in the kinetic response to vaccination and infection in our cohort because of this missing information. Additionally, the absence of consistent post-vaccination timepoints for all participants likely increased variation in our study data. It is also important to note that we did not measure the antibody neutralizing capacity or cellular immune characteristics in the study cohort. We used the anti-RBD monoclonal antibody CR3022 to establish a standard curve to ascertain the IgG concentrations in participant serum as previously described (20–22). To control for the possibility of reduced sensitivity of the ELISA assay due to potential lower affinity of mAb CR3022, we validated mAb CR3022 to ensure its sensitivity and specificity using stringent washing conditions and a short 2-hour incubation time. Parallel tests were performed using a human IgG reference protein from plasma, which showed comparable results.

In conclusion, our study shows that in a cohort of high risk-individuals, occupational exposure to SARS-CoV-2 and vaccination response varied. However, this variation was not based on occupational exposure risk, as healthcare providers and non-providers exhibited equivalent outcomes. Further, not all infected participants developed a serological response to the NP protein of SARS-CoV-2. As such, a non-trivial proportion of participants had serological evidence of infection without their knowledge based on reporting symptoms. The humoral response was greater and more stable in response to the full SARS-CoV-2 S protein as compared to the RBD, the latter being the target of existing vaccines. Demographically, Hispanic participants are at a higher risk of breakthrough infection than non-Hispanic participants among vaccinated individuals in this cohort of high-risk frontline workers at an urban medical center community in southern Los Angeles County. Finally, the antibody response to RBD was not predictive of breakthrough infection after vaccination or before infection.

## Data availability statement

The raw data supporting the conclusions of this article will be made available by the authors, without undue reservation.

## Ethics statement

The studies involving human participants were reviewed and approved by the Institutional Review Board of the University of California, Los Angeles (IRB#20-001649). The patients/participants provided their written informed consent to participate in this study.

## Author contributions

MJ performed the experiments, analyzed the data, and wrote the manuscript. HP contributed data analysis and validated the initial assay used in this study. YZ and DG significantly contributed to statistical analyses. DP and EF are clinical coordinators/phlebotomists who recruited all subjects and directed patient interactions, sample collections, and input the data into RedCap. DK designed the surveys and reviewed of all clinical data collection elements. TR provided necessary proteins for the ELISA assays and reviewed the manuscript. JS, LM, MY, and ER designed the study, significantly contributed to data interpretation and revised the manuscript. All authors contributed to the article and approved the submitted version.
