# Supplementary material for: Longitudinal analysis of SARS-CoV-2 infection and vaccination in the LA-SPARTA cohort reveals increased risk of infection in vaccinated Hispanic participants
Source: Front Immunol. 2023 Apr 19;14:1139915. doi: 10.3389/fimmu.2023.1139915 (PMC10154521; doi:10.3389/fimmu.2023.1139915)
Supplement: Supplementary file 1 [file DataSheet_1.pdf]

Supplementary Material

Longitudinal analysis of SARS-CoV-2 infection and vaccination in the LA-SPARTA cohort reveals increased risk of infection in vaccinated Hispanic participants

Meagan M. Jenkins[1], Donna Phan Tran[2], Evelyn A. Flores[2], Deborah Kupferwasser[2], Harry Pickering[1], Ying Zheng[1], David W. Gjertson[1,3,] Ted M. Ross[4,5,] Joanna M. Schaenman[6,7†] Loren G. Miller[2,6,8,7†] Michael R. Yeaman[6,8-10†] Elaine F. Reed1[* †]

*** Correspondence:** Elaine F. Reed: ereed@mednet.ucla.edu

1 Supplementary Figures and Tables

1.1 Supplementary Figures

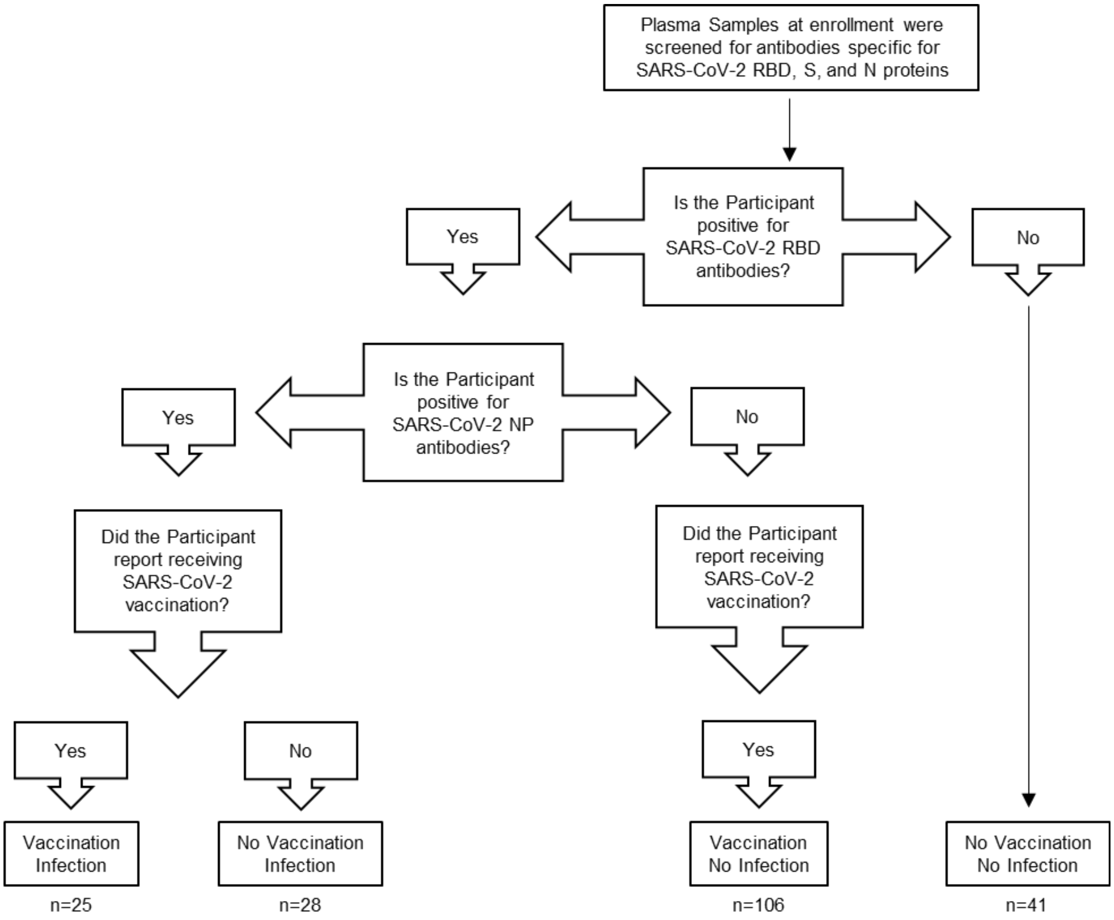

Supplemental Figure 1. Flow Chart of participant grouping at enrollment based on serology. The participant's plasma was tested for anti-SARS-CoV-2 S, RBD, and NP antibodies, and grouped based on RBD and NP responses at enrollment. This flow chart outlines how the participants were grouped based on this.

Supplemental Figure 2

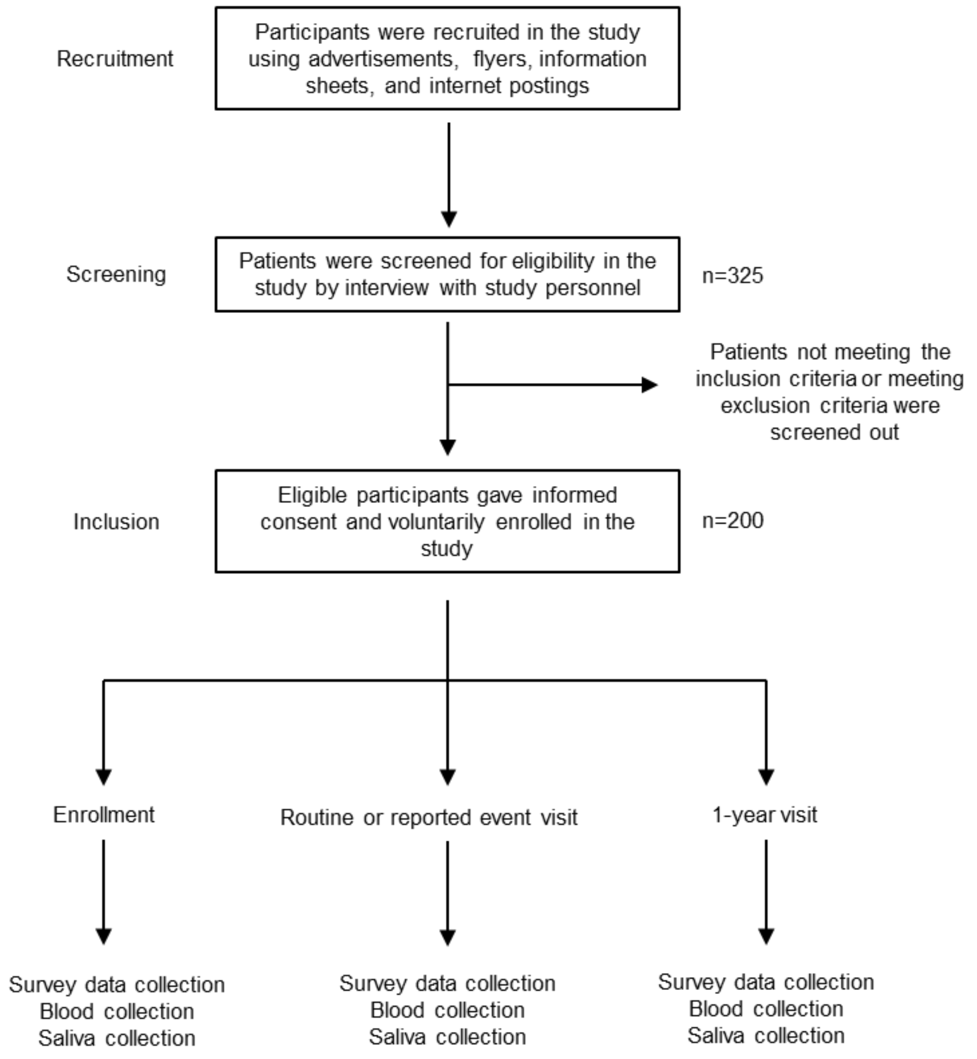

Supplemental Figure 2. Study Design Flow Chart. This figure shows the work flow of participant recruitment, screening, inclusion, enrollment, and sample collection.
